# Successful Combination Therapy With Lenvatinib Plus Pembrolizumab for Rare Case of Advanced Renal Cell Carcinoma With Ocular Adnexal Metastasis

**DOI:** 10.1002/cnr2.70437

**Published:** 2025-12-21

**Authors:** Yozo Mitsui, Masato Uetani, Fumito Yamabe, Hideyuki Kobayashi, Aki Mitsuda, Naobumi Tochigi, Koichi Nakajima

**Affiliations:** ^1^ Department of Urology Toho University Faculty of Medicine Tokyo Japan; ^2^ Department of Surgical Pathology Toho University Faculty of Medicine Tokyo Japan

**Keywords:** immuno‐oncology therapy, metastatic renal cell carcinoma, ocular adnexal metastases, tyrosine kinase inhibitor

## Abstract

**Background:**

Ocular adnexal (OA) metastasis from renal cell carcinoma (RCC) is a very rare end‐stage entity with a poor prognosis. Reported here is a case of metastatic RCC for which combination therapy with lenvatinib plus pembrolizumab was given for OA metastasis and achieved an excellent response.

**Case:**

A 54‐year‐old man was referred to our hospital complaining of right eye pain, with a right intraocular tumor showing a maximum diameter of 3 cm subsequently revealed by computed tomography (CT) imaging. Additionally, masses in the right kidney, both lungs, and right iliac bone were noted, while a subsequent percutaneous renal needle biopsy indicated RCC with multiple metastases (cT3aN0M1). Administration of lenvatinib plus pembrolizumab combination therapy was given as first‐line treatment, with rapid improvement of the elevated inflammatory response and anemia noted. Findings obtained 17 months following initiation of therapy showed significant shrinkage of the primary and iliac bone lesions, while the OA and lung metastatic lesions had completely disappeared.

**Conclusion:**

These results highlight the potential of lenvatinib plus pembrolizumab combination therapy for the treatment of RCC with OA metastasis, which is generally considered to be an end stage for cancer cases.

## 1 Introduction

Renal cell carcinoma (RCC) is one of the most common urological cancers, with approximately one‐third of affected patients presenting metastatic disease at the time of diagnosis [1]. The most common sites are the lungs, bone, liver, and brain, while extremely rare advanced disease cases showing metastasis to the ocular adnexa (OA) or intraocular space have also been reported [1, 2]. Although recent advances in diagnostic methods and targeted therapy have contributed to improved survival in cancer patients with OA metastasis, they generally have a poor prognosis [2, 3].

Presently, immune checkpoint inhibitor (ICI)‐based combination regimens are recognized as a promising standard first‐line treatment for advanced RCC, including metastatic disease. Among those, the CLEAR trial showed promising efficacy with the combination of pembrolizumab, an ICI targeting programmed cell death protein‐1 (PD‐1), plus lenvatinib, a multitargeted tyrosine kinase inhibitor (TKI), for treating metastatic RCC [4]. More recently, real‐world efficacy and safety of this regimen for advanced RCC patients were also confirmed in a Japanese population [5]. Presented here is the first case report of advanced RCC with OA metastasis successfully treated with lenvatinib plus pembrolizumab combination therapy.

## 2 Case Presentation

**FIGURE 1:**
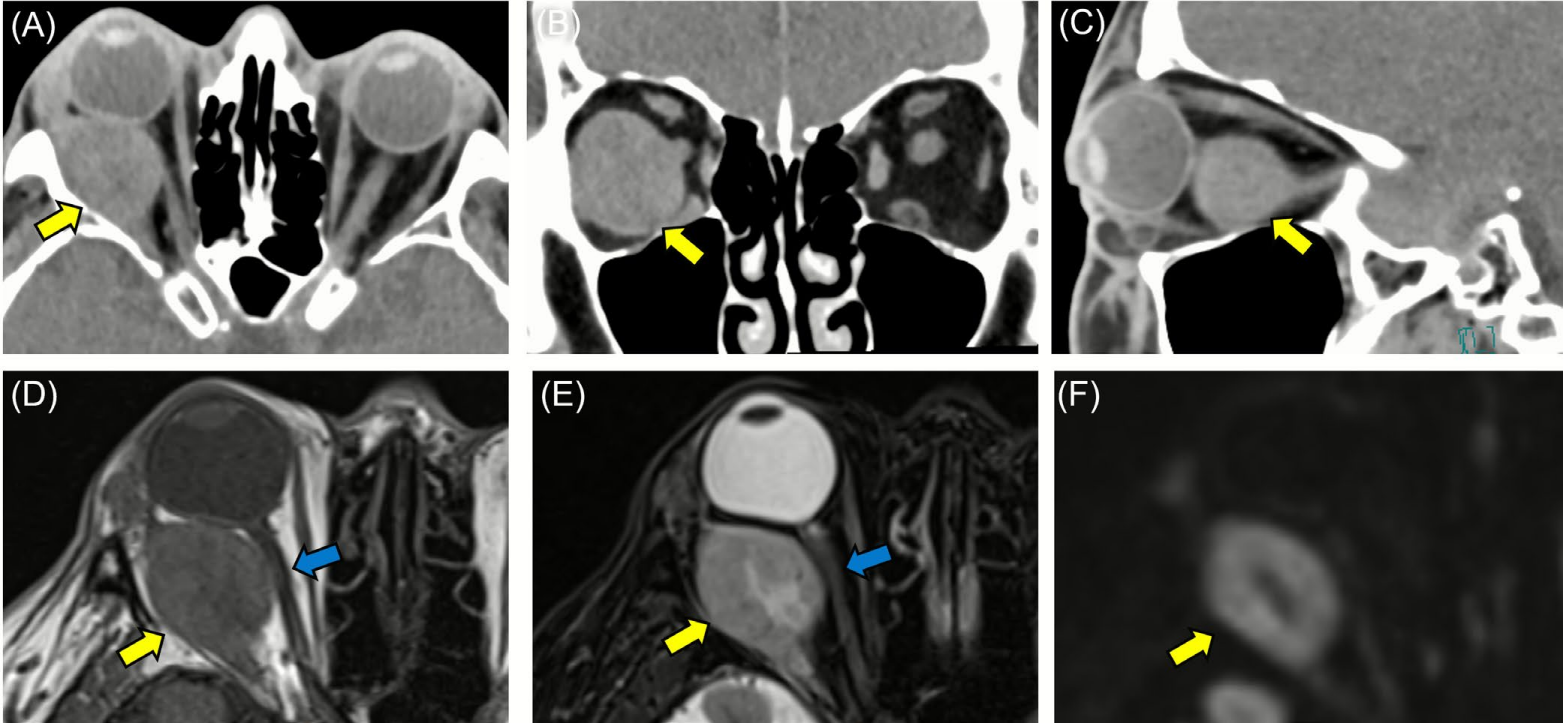
Imaging findings showing ocular adnexal metastasis from renal cell carcinoma. Head computed tomography scanning of the right orbital tumor lesion before treatment showed a round mass measuring 3.0 × 2.4 × 2.7 cm in the posterior‐lateral area of the right eyeball (A), axial section; (B), coronal section; (C), transverse section. This right orbital mass showed a slightly low signal level in magnetic resonance imaging T1 weighted images (WI) (D), a signal level similar to that of muscle tissue in T2WI (E), and a high signal level in diffusion weighted images (F). Yellow and blue arrows indicate the right orbital mass and right optic nerve, respectively.

**FIGURE 2:**
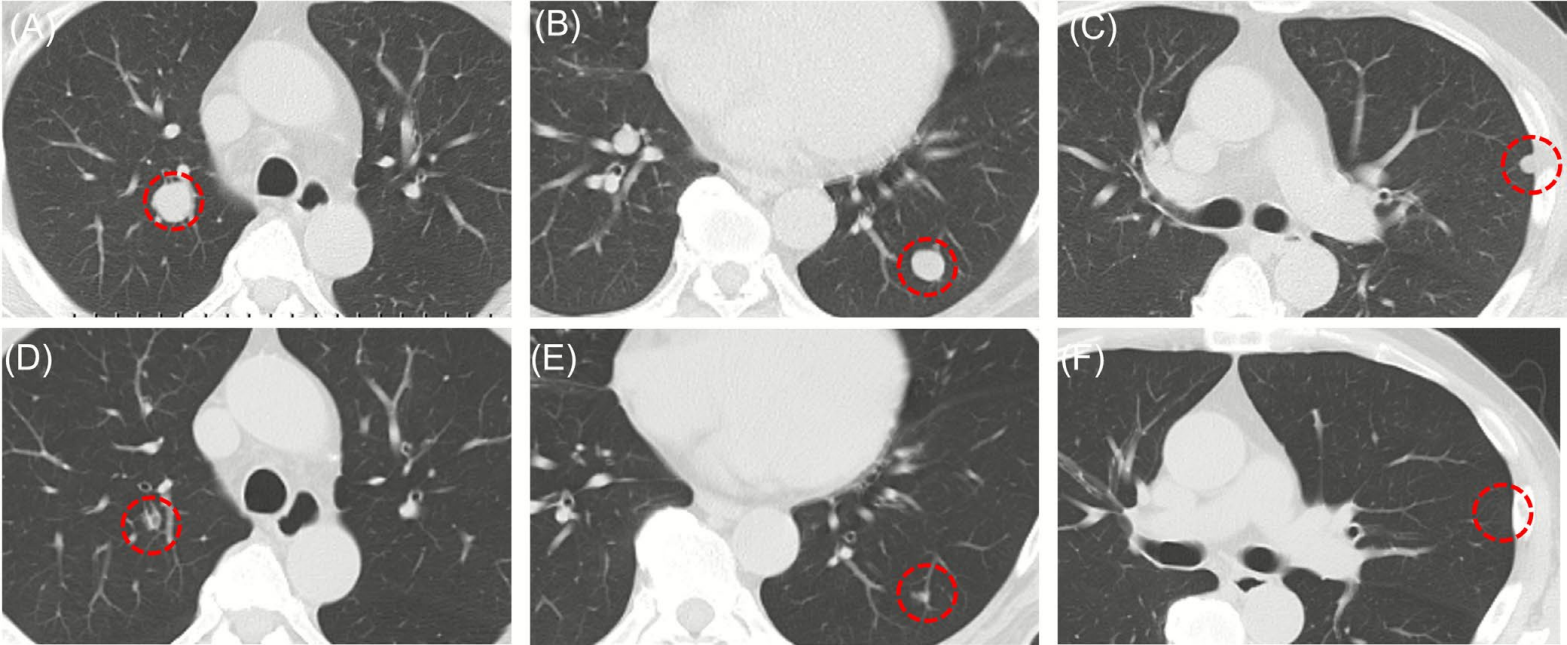
Imaging findings showing pulmonary metastatic lesions before and after anticancer treatment. Multiple metastases were confirmed in both lungs (A–C) before initiating combination therapy with lenvatinib plus pembrolizumab. Those had completely disappeared after 17 months of treatment (D–F). Red dash circles indicate metastatic lesions or areas where metastatic lesions were present.

**FIGURE 3:**
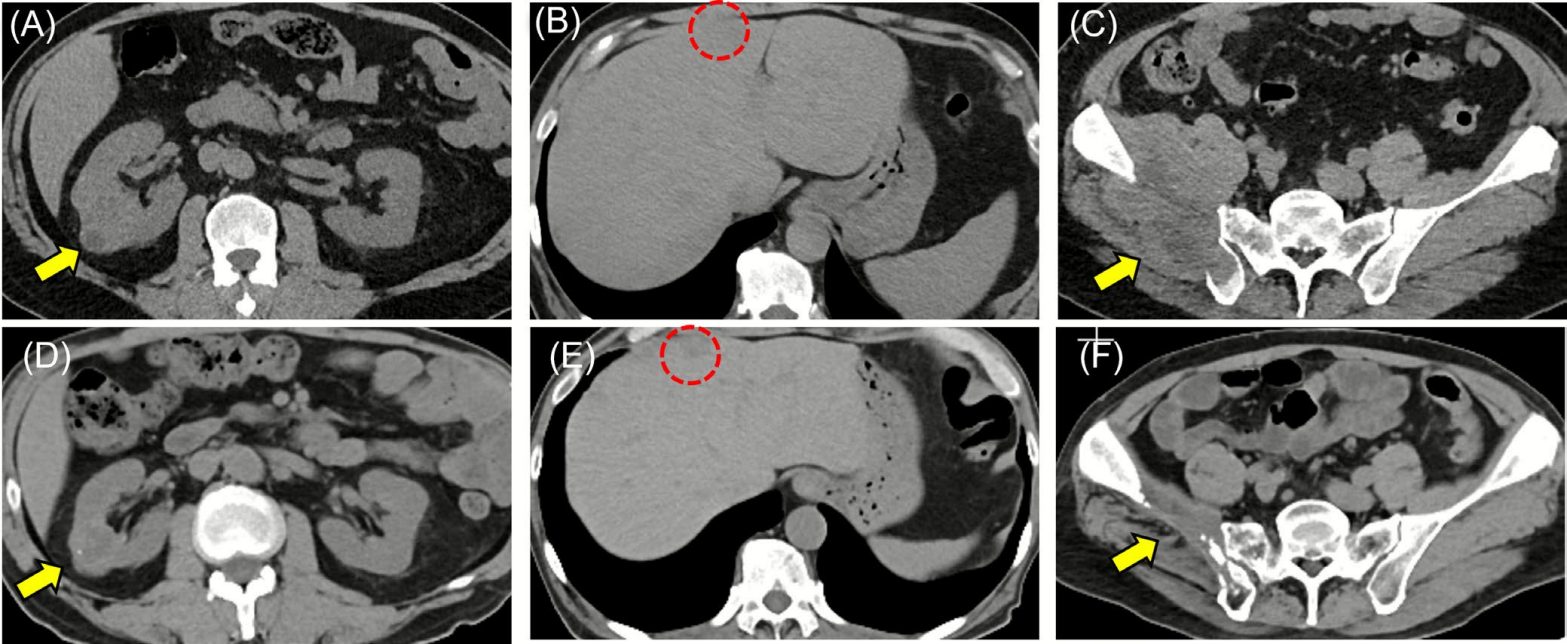
Imaging findings showing primary lesions and liver and bone metastases before and after anticancer treatment. Prior to initiation of lenvatinib plus pembrolizumab therapy, existence of a primary right kidney tumor (A), liver metastasis (B), and right iliac metastasis (C) was confirmed. Maintained partial response was noted at 17 months after the start of treatment (D–F). Yellow arrows indicate primary and red dashed circles indicating metastatic lesions.

**FIGURE 4:**
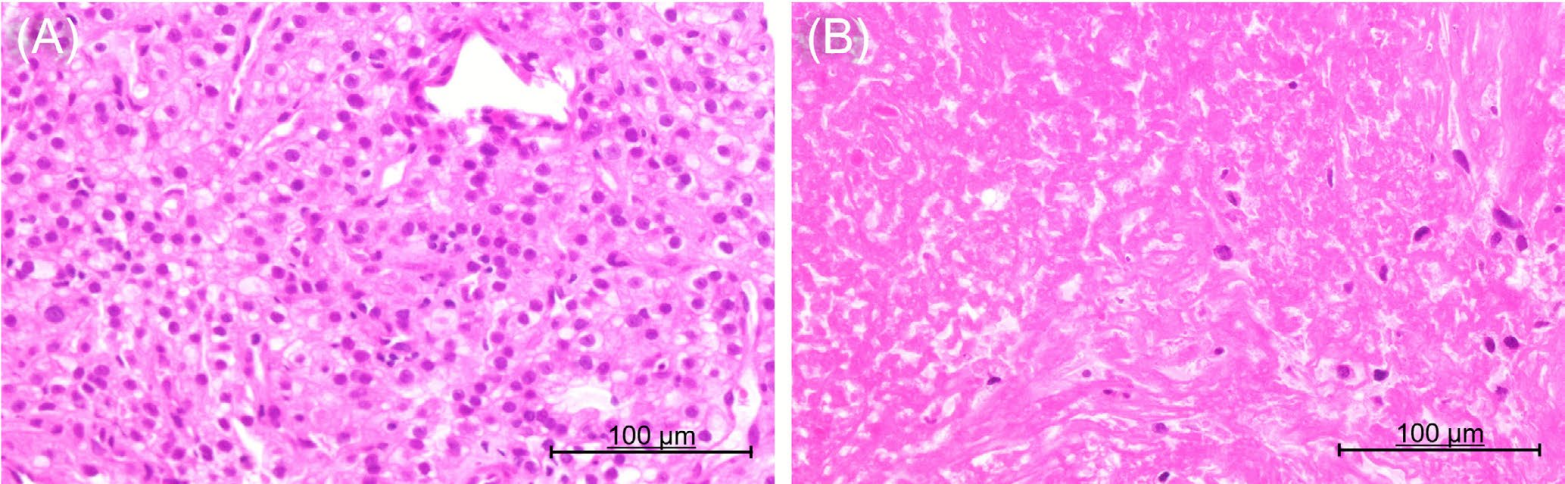
Pathological findings of renal tumor tissue sample obtained by percutaneous needle biopsy. Hematoxylin and eosin staining of biopsied tissue showed clear cell RCC, including nests of atypical cells with round nuclei and clear or pale eosinophilic cytoplasm (A). Extensive necrotic tissue around the tumor was also observed (B).

A 54‐year‐old man was referred to the ophthalmology department of our medical center from a nearby hospital because of visual impairment and pain in the right eye that had continued for about 2 months. At the initial consultation, posterior scleritis of the right eye was suspected, while subsequent head computed tomography (CT) findings revealed a tumorous lesion in the right orbit (Figure 1A–C). The only condition noted in the medical history was asthma; thus, contrast medium could not be used and plain magnetic resonance imaging (MRI) of the head was performed to evaluate the right orbit lesion in detail. Those findings confirmed a round‐shaped mass sized 3.0 × 2.4 × 2.7 cm on the posterior lateral side of the eyeball compressing the optic nerve from the outside, which was thought to be a tumor originating from the lateral rectus muscle (Figure 1D–F). The patient was anemic and showed elevated inflammatory markers; thus, based on the possibility of a primary or metastatic orbital malignant tumor, whole‐body plain CT scanning was performed. The results confirmed multiple masses in the lungs, while tumorous lesions were also noted in the right kidney, liver, and right ilium (Figures 2 and 3). Additional plain MRI findings of the abdominal region revealed a 6 cm mass in the right kidney with irregular margins and hemorrhaging, as well as a heterogeneous high signal in diffusion‐weighted images; thus, a right renal cell carcinoma was suspected as the primary disease (data not shown). The patient was admitted to our hospital and underwent a percutaneous needle biopsy of the right renal tumor for determining diagnosis. Histopathological findings of the biopsy specimen indicated clear cell RCC accompanied by necrosis (Figure 4), and the patient was ultimately diagnosed with a multiple metastatic RCC (cT3aN0M1), including right OA metastasis. No genetic or molecular examinations including testing for PD‐L1 expression were performed for the present case.

**FIGURE 5:**
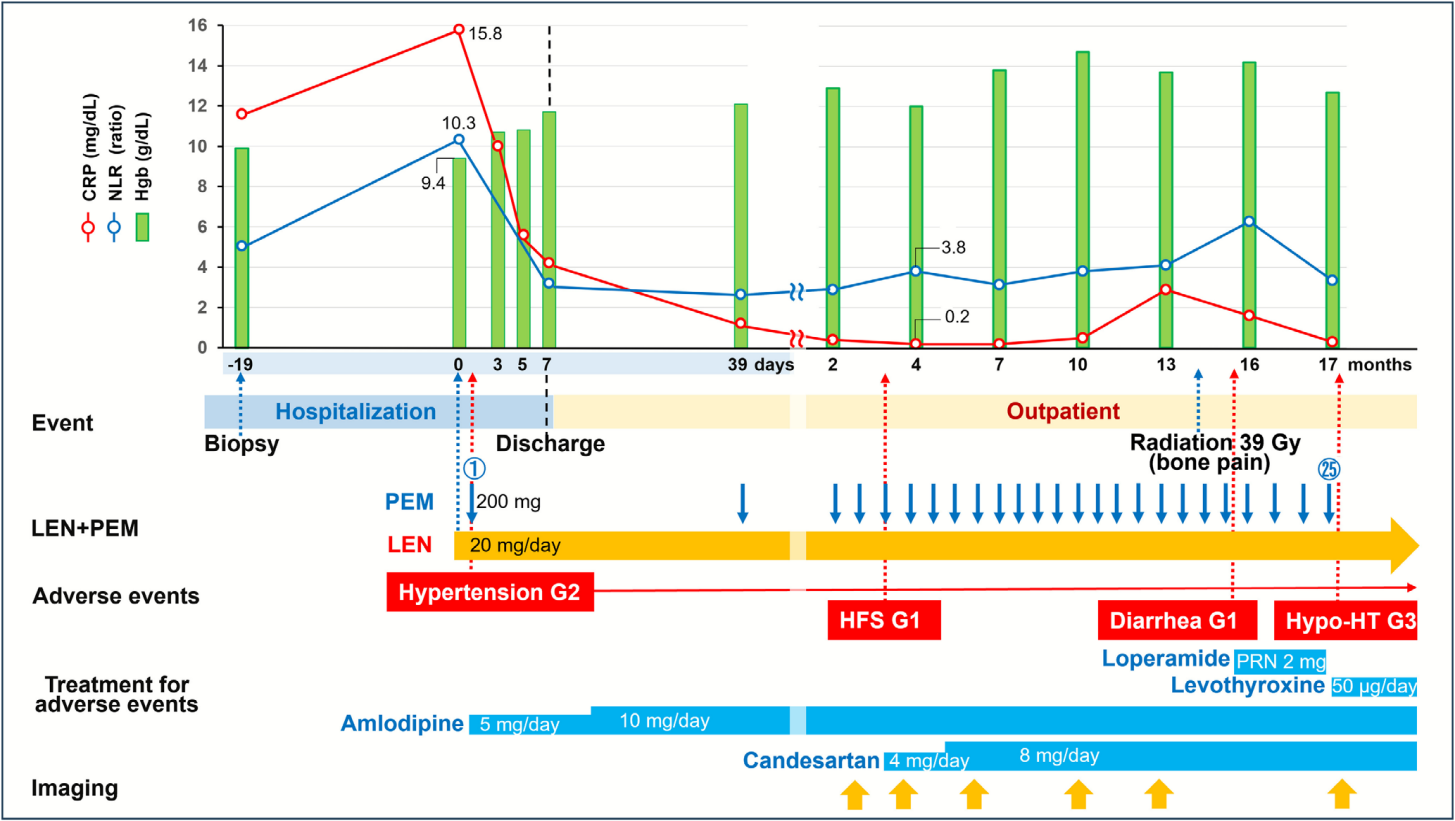
Clinical course. Following diagnosis of multiple metastatic renal cell carcinoma (cT3aN0M1) with ocular adnexal metastasis, combination therapy with lenvatinib (20 mg/day) plus pembrolizumab (200 mg/3 weeks) was initiated. After treatment initiation, anemia rapidly improved, and the C‐reactive protein and neutrophil/lymphocyte ratio decreased. At the time of writing, treatment has been maintained for 17 cycles without interruption. Complete response was achieved after 17 months for the right OA metastasis and bilateral multiple lung metastases, while partial response was maintained for the primary cancer lesion in the right kidney, and liver and right iliac metastases. CRP, C‐reactive protein; NLR, neutrophil/lymphocyte ratio; LEN, lenvatinib; Pem, pembrolizumab; HFS, hand‐foot syndrome; Hypo‐HT, hypothyroidism; PRN, pro re nata.

The clinical course following admission is shown in Figure 5. Pretreatment evaluation findings classified the patient as poor risk, as four of the six risk factors of the International Metastatic RCC Database Consortium (IMDC) classification were present: time from diagnosis to systemic therapy within 1 year, low hemoglobin level, and high neutrophil and platelet counts. Upfront removal of the primary RCC prior to systemic treatment was proposed; however, the patient did not wish to undergo surgery. Aiming for a rapid and high therapeutic effect on the metastatic RCC, lenvatinib plus pembrolizumab combination therapy was administered as first‐line treatment from 19 days after the biopsy. The starting doses of lenvatinib and pembrolizumab were 20 mg/day and 200 mg/3 weeks, respectively. Immediately after starting systemic therapy, the patient showed grade 2 hypertension, though that was manageable with antihypertensive medication, and the starting doses were maintained. Anemia (hemoglobin 9.4 g/dL) also noted in the patient prior to starting systemic therapy improved soon thereafter. Similarly, both pre‐treatment C‐reactive protein (CRP) and neutrophil/lymphocyte ratio (NLR), 15.8 mg/dL and 10.3, respectively, rapidly decreased after the start of systemic therapy to negative and < 4, respectively, after 4 months of treatment. On day 7 following initiation of lenvatinib plus pembrolizumab combination therapy, disappearance of right ocular pain and dramatic recovery of vision were noted, and the patient was discharged.

Lenvatinib plus pembrolizumab was continued as outpatient care, with the patient visiting every 3 weeks. A rise in blood pressure was noted during that period, thus the dose of antihypertensive medication was increased, while angiotensin II receptor antagonist administration was later added for cardioprotection and antihypertensive purposes. While the patient experienced grade 1 hand‐foot syndrome, diarrhea, and grade 2 hypothyroidism, no serious adverse events were observed and the starting doses of both drugs remained the same. At the time of writing, treatment has been maintained for 17 cycles without interruption.

**FIGURE 6:**
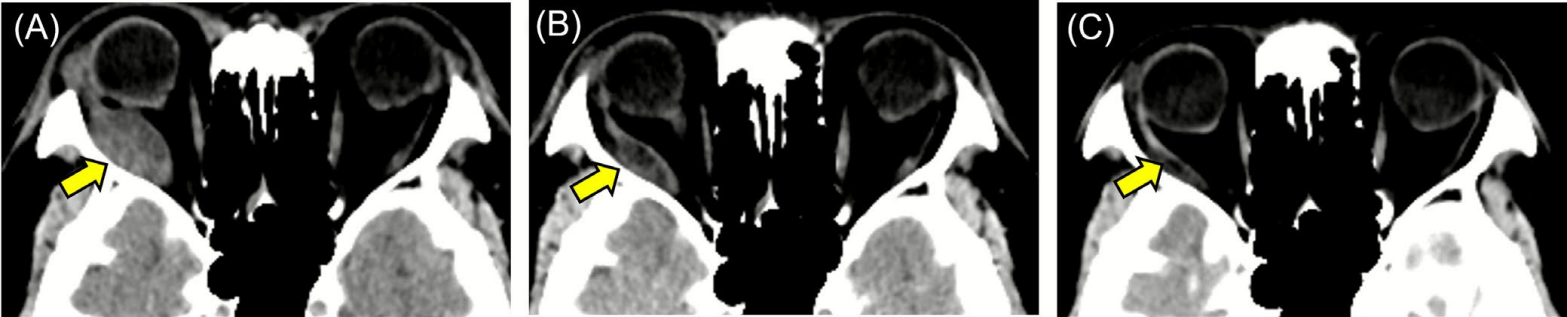
Longitudinal changes noted in imaging findings of ocular adnexal metastasis during treatment. After initiation of lenvatinib plus pembrolizumab therapy, the area of metastasis in the right ocular adnexal area showed dramatic shrinkage after two (A) as well as 12 (B) months, with complete response confirmed after 17 months (C). Yellow arrows indicate right orbital metastasis.

During the treatment course, the therapeutic effects were periodically evaluated by whole‐body plain CT scanning. Notably, a dramatic reduction in size of the right OA metastasis was found after three treatment cycles in the first 2 months and then was further reduced after 12 months, with complete disappearance after 17 months (Figure 6). Finally, the conjunctival abnormalities and right ocular motility disorder observed at the initial consultation completely resolved, and there were no residual complications due to optic nerve compression by the tumor. Also, multiple metastatic lesions identified in both lungs showed complete response (CR) at 17 months after starting lenvatinib plus pembrolizumab combination therapy (Figure 2D–F). Furthermore, the primary cancer lesion in the right kidney and metastatic lesion in the liver both showed decreased size over time and were judged as partial response (PR) to treatment after 17 months (Figure 3D,E). Regarding the right iliac bone metastasis, external radiation therapy was performed for mild tumor growth accompanied by pain noted during the course of treatment. However, tumor shrinkage was noted at 17 months and the treatment effect was determined to be PR (Figure 3F). The results indicated that lenvatinib plus pembrolizumab combination treatments maintained a high therapeutic effect for the present patient; thus, those are currently ongoing along with whole‐body CT scans performed every 3 months.

## 3 Discussion

The orbit is a rare site of cancer metastasis, with incidence ranging from 1% to 13% of all orbital tumor cases [6]. The main primary tumors leading to orbital metastasis are related to lung cancer for intraocular and breast cancer for OA, while renal cell carcinoma is not often involved, accounting for only 5% to 11% of orbital metastasis cases [7]. A definitive diagnosis of OA metastasis from RCC essentially requires a tissue biopsy procedure, though empirical diagnosis based on clinical and imaging findings may also be possible [2]. Typical symptoms of OA metastasis are those related to the mass effect, such as proptosis, mass, swelling, and diplopia. The orbital imaging modality that plays the most important role for diagnostic assessment of OA metastases is CT scanning, performed alone or in combination with other imaging modalities [2]. CT images show OA metastatic lesions as solitary masses with irregular borders, which are occasionally accompanied by bone destruction, intracranial extension, or extension to the paranasal sinuses. For the present case, diagnosis of OA metastasis from RCC was based on RCC biopsy results, as well as clinical and imaging findings. In general, orbital metastasis is associated with poor overall prognosis, with a retrospective study of 42 cases of such metastasis from any primary site showing a 5‐year overall survival (OS) rate of 7% [3]. On the other hand, results of a recent study that reviewed cases of OA metastasis from RCC between 1990 and 2020 showed that OS for patients with this disease has gradually improved since new treatments have become available, such as TKIs for metastatic RCC [2]. Nevertheless, that study found that even with multimodality treatments using combinations of TKIs, OS improvement for RCC patients with metastasis to other sites in addition to OA was significantly lower as compared to patients with OA metastasis alone. Therefore, treatment for this condition remains challenging.

The advent of ICIs for use as therapeutic agents has led to a paradigm shift regarding management of progressive and metastatic RCC. In particular, combinations of TKIs and ICIs now play an important role in immuno‐oncology therapy for advanced RCC, and such treatment is considered to surpass the therapeutic benefits of TKI monotherapy. An international joint phase III study (CLEAR study) confirmed that pembrolizumab plus lenvatinib therapy, a TKI and ICI combination, has high therapeutic efficacy for metastatic RCC and became available in Japan in 2022 [4]. Another study examined treatment outcomes of 50 patients in Japan with advanced RCC who received combination therapy with pembrolizumab plus lenvatinib and found that the objective response rate was achieved in around 60% regardless of IMDC risk, indicating that this treatment is promising for a Japanese population [5]. OA metastasis from RCC is a late‐stage disease that occurs on average 6 to 7 years after diagnosis of the primary tumor [3]. However, in the present case of metastatic RCC, OA metastasis was noted at the time of diagnosis and considered to have a high level of malignancy and potential for rapid progression. Therefore, lenvatinib plus pembrolizumab was used as first‐line treatment and expected to have anticancer effects. Notably, CR was obtained by treating OA metastasis, thus demonstrating a strong anticancer effect against primary tumors and other metastatic lesions. In a previous study that included cases other than OA without metastatic lesions, suppression of disease progression by treatment was found in less than 30% of the affected patients [2]. To the best of our knowledge, no other cases of immuno‐oncology therapy performed for advanced RCC with OA metastasis have been reported, indicating that this is the first presentation of findings showing a good therapeutic effect achieved with administration of pembrolizumab plus lenvatinib as immunotherapy.

Among TKIs used in combination with ICIs for advanced RCC, lenvatinib has selective inhibitory activities mainly towards vascular endothelial growth factor (VEGF) and fibroblast growth factor (FGF) receptors, thus tumor angiogenesis and malignant progression are strongly suppressed by inhibiting both pathways. In RCC cases, FGF receptors are highly expressed in metastatic lesions, such as those occurring in lymph nodes, as well as primary tumors [8]. Interestingly, a previous study noted that inhibition of the FGF pathway significantly reduced the angiogenic ability of RCC bone metastatic cell lines [9]. Together, these findings provide a scientific rationale for the excellent therapeutic effects of lenvatinib toward metastatic lesions as well as RCCs. Pembrolizumab is an antibody against PD‐1 that activates CD8+ T cells by inhibiting PD‐1 binding to its ligand, thereby exerting an antitumor effect. Recent studies have shown that activation by RCC of the VEGF pathway suppresses stimulation of CD8+ T cells by activating tumor‐associated macrophages (TAMs) and inhibiting the interferon (IFN) signaling pathway [10, 11]. Additionally, those studies noted that lenvatinib can regulate cancer immunity in the tumor microenvironment by reducing TAMs and enhance the antitumor effect of anti‐PD‐1 antibodies through activation of the IFN signaling pathway. Importantly, the enhancing effect of anti‐PD‐1 antibodies on antitumor activity was reported to be attenuated when axitinib, another TKI without an inhibitory effect on FGF receptors, was administered in place of lenvatinib [11]. Therefore, the high antitumor effect of lenvatinib plus pembrolizumab combination therapy, also noted in the present case, is likely due to their beneficial interactions as well as the effects of each drug alone. There are several other first‐line combination systemic therapies available for advanced RCC, each of which exerts a high therapeutic effect due to drug interactions. Although it was only an indirect comparison, results of a recent Bayesian network meta‐analysis suggest that the combination of lenvatinib and pembrolizumab has a therapeutic effect greater than other approved immune checkpoint inhibitor combination therapies [12]. Those findings strongly influenced selection of the combination of lenvatinib and pembrolizumab as first‐line treatment for the present case.

The prognostic predictive ability of inflammatory markers, such as CRP and NLR, in metastatic RCC cases is well known [13, 14]. Recent research in this field has focussed on the relationships of changes in these inflammatory markers with clinical outcomes of immuno‐oncology therapy. Schüttke et al. found that normalization of CRP within 3 months after treatment reflects good prognosis for patients with metastatic RCC undergoing first‐line therapy with an ICI [15]. Furthermore, a study conducted by Nakayama et al. showed that over the course of treatment NLR as well as CRP kinetics were useful biomarkers for predicting prognosis of RCC patients undergoing immuno‐oncology therapy [16]. Based on their findings, they proposed that when NLR is 4.8 or higher and/or CRP is 1.0 mg/dL or higher at approximately 2 months after treatment, continuation of the treatment should be reconsidered. As for the mechanism underlying those findings, CRP and NLR levels may reflect the tumor‐promoting inflammatory microenvironment and immune system balance in malignant tumors. Regarding NLR, Hwang et al. [17] found that T cell proliferation induced by increased interferon‐γ secretion by immunotherapy leads to a decrease in NLR in non‐small cell lung cancer. Additionally, that study showed that the decrease in NLR level reflects a therapeutic effect and is associated with a good prognosis following immunotherapy [17]. It is thus considered that these inflammatory biomarkers are useful for predicting response and prognosis of patients undergoing immuno‐oncology therapy for RCC, since fluctuations of CRP and NLR values associated with treatment response state were also noted in the present case.

In the CLEAR study, the discontinuation rate due to adverse events (AEs) in the lenvatinib plus pembrolizumab combination therapy group was 36%, higher as compared to the control group at 11% [4]. It will be necessary to fully understand the characteristics of AEs caused by this therapy and how to treat them, as appropriate management of those associated with cancer therapy is important for treatment continuation. Fortunately, no severe AEs have been noted in the present patient, though close attention to possible occurrence will be given as treatment continues. Currently, while taking into consideration the importance of balance between therapeutic effects and quality of life, should no AE occur, a reduction in the dosage of one or both drugs, or even temporary discontinuation is available options.

In conclusion, reported here is the first case of multiple metastatic RCC, including OA metastasis, treated with lenvatinib plus pembrolizumab as combined onco‐immunotherapy. This combination has shown promising results and can be expected to have a high therapeutic effect, even for advanced RCC with OA metastasis, known as a difficult condition to manage. Nevertheless, the present report is based on findings of a single patient, thus accumulation of additional cases and related research of biomarkers in regard to treatment selection are necessary to confirm these results.

## Author Contributions

**Yozo Mitsui:** conceptualization, methodology, investigation, data curation, resources, writing – original draft, writing – data curation, writing – review and editing, project administration. **Masato Uetani:** conceptualization, methodology, investigation. **Fumito Yamabe:** conceptualization, data curation. **Hideyuki Kobayashi:** methodology, data curation. **Aki Mitsuda:** investigation, visualization. **Naobumi Tochigi:** investigation, visualization. **Koichi Nakajima:** resources, supervision.

## Funding

The authors have nothing to report.

## Ethics Statement

All procedures followed in this study were performed in accordance with the ethical standards laid down in the 1964 Declaration of Helsinki and its subsequent amendments.

## Consent

Informed consent was obtained from the present patient for publication of this study.

## Conflicts of Interest

The authors declare no conflicts of interest.

## Data Availability

The data that support the findings of this study are available from the corresponding author upon reasonable request.
